# 离子排斥色谱法同时测定复方氨基酸注射液（14AA）中甘油、醋酸及亚硫酸氢钠

**DOI:** 10.3724/SP.J.1123.2026.01005

**Published:** 2026-07-08

**Authors:** Yongsheng GU, Limin ZUO, Xiaoli GAO, Guangzhi SHAN, Jun ZHAO

**Affiliations:** 1. 新疆医科大学药学院，新疆 乌鲁木齐 830017; 1. College of Pharmacy，Xinjiang Medical University，Urumqi 830017，China; 2. 中国医学科学院医药生物技术研究所，北京 100050; 2. Institute of Medicinal Biotechnology，Chinese Academy of Medical Sciences，Beijing 100050，China; 3. 新疆及中亚特色医药资源教育部工程研究中心，新疆 乌鲁木齐 830054; 3. Engineering Research Center of Xinjiang and Central Asian Medicine Resources，Ministry of Education，Urumqi 830054，China; 4. 新疆医科大学第一附属医院药学部，新疆 乌鲁木齐 830011; 4. Pharmacy Department of the First Affiliated Hospital of Xinjiang Medical University，Urumqi 830011，China

**Keywords:** 离子排斥色谱法, 复方氨基酸注射液（14AA）, 甘油, 醋酸, 亚硫酸氢钠, ion exclusion chromatography, compound amino acid injection （14AA）, glycerol, acetic acid, sodium bisulfite

## Abstract

采用离子排斥色谱法，建立了同时测定复方氨基酸注射液（14AA）中甘油、醋酸及亚硫酸氢钠的方法。色谱柱采用Xtimate Sugar‐H柱（300 mm×7.8 mm，5 μm），流动相为7.5 mmol/L 硫酸溶液，等度洗脱，流速为0.5 mL/min，检测波长200 nm，柱温为65 ℃，进样量为10 μL。以紫外检测器进行检测，外标法定量。结果表明，本方法专属性良好，氨基酸成分不干扰甘油、醋酸及亚硫酸根测定，主峰与相邻色谱峰分离度良好，甘油、醋酸及亚硫酸根在一定范围内线性关系良好，相关系数（*r*）均>0.999 5。针对3种成分进行了低、中、高3个水平的加标回收试验，目标物的平均回收率为99.21%~102.6%，相对标准偏差（RSD，*n*=3）为0.09%~0.86%。该方法能够有效解决氨基酸成分的干扰，快速简便，专属性强，准确度高，可用于复方氨基酸注射液（14AA）中甘油、醋酸及亚硫酸氢钠的含量测定。

## Abstract (en)

An ion exclusion chromatography method was established for the simultaneous determination of glycerol， acetic acid and sodium bisulfite in compound amino acid injection （14AA）. No tedious sample pretreatment is required； the sample can be directly injected after filtration through a 0.22 μm membrane filter. Chromatographic separation was achieved using an Xtimate Sugar-H column （300 mm×7.8 mm， 5 μm） packed with a strong ion-exchange resin based on a rigid styrene/divinylbenzene matrix， which is specially designed for the separation of sugar alcohols and organic acids via the ion-exclusion mechanism. The mobile phase was 7.5 mmol/L sulfuric acid solution， which was selected as it can suppress the ionization of target analytes and enhance their retention on the stationary phase； the amino groups of amino acid components undergo strong ionization， leading to no retention. Isocratic elution was adopted to ensure stable separation efficiency and short analysis time， with the flow rate set at 0.5 mL/min. Based on the ultraviolet absorption characteristics of the target analytes， 200 nm was selected as the detection wavelength. The column temperature was set at 65 ℃ and the injection volume was optimized to 10 μL， which ensures the detection sensitivity while avoiding column overload. The external standard method was adopted for quantitative analysis， featuring simple operation and reliable results. The method exhibited excellent specificity： blank solvent and negative matrix solution tests verified that amino acids caused no interference with the determination of target analytes， and the target analytes achieved good resolution from adjacent peaks. For the linearity investigation， standard solutions were prepared with linear ranges set as follows： 1.197–59.87 mg/mL for glycerol， 0.036 08–1.804 mg/mL for acetic acid， and 0.000 791 6–0.633 3 mg/mL for sulfite. Within these mass concentration ranges， the linear correlation coefficients （*r*） of all components were greater than 0.999 5， indicating an excellent linear relationship of the method. The accuracy was verified by spiked recovery tests at three levels （80%， 100% and 120% of the labeled amount）， with three parallel preparations for each level. The results showed that the average recoveries of the target analytes ranged from 99.21% to 102.6%， with relative standard deviations （RSDs） of 0.09% to 0.86% （*n*=3）， demonstrating the high accuracy of this method. The ion exclusion chromatography method established in this study can effectively eliminate the interference of the amino acid matrix in compound amino acid injection （14AA）， and has the advantages of rapid analysis， simple operation， strong specificity and high accuracy， making it suitable for the routine quality control of glycerol， acetic acid and sodium bisulfite in this preparation. This method provides a reliable analytical and testing tool for the quality assurance and safety control of compound amino acid injection （14AA） during production and storage.

复方氨基酸注射液（14AA）是一种平衡型肠外营养制剂，由14种氨基酸、甘油及抗氧剂配制而成，主要用于改善手术前后病人的营养状态，纠正蛋白质消化吸收障碍及轻度营养不良^［1］^。甘油作为人体生理需要的热量源，可提供弱酸性环境，有助于氨基酸稳定及代谢优化，从而提升其利用率。醋酸主要来源于制剂处方中的赖氨酸醋酸盐，此外，制剂过程中因调节pH值而引入的醋酸，也是其来源之一；其含量的高低会影响注射液的pH值，进而可能影响氨基酸降解、聚合以及药液的澄明度和渗透压等^［2］^。亚硫酸氢钠作为常用的抗氧化剂，可抑制处方中易氧化氨基酸降解产生相关杂质^［3，4］^。

目前，该制剂在各国药典中均未收载，现行标准执行国家药品标准WS1-（XG-005）-2020，但未对甘油、醋酸及亚硫酸氢钠进行质控。现行中国药典标准中，甘油原料药采用高碘酸钠氧化滴定法测定，醋酸和亚硫酸氢钠作为药用辅料，分别采用酸碱滴定法和氧化还原滴定法检测^［5］^，然而，这些滴定法均为针对原料药或单一辅料，存在操作繁琐、易受其他成分干扰，灵敏度较低等局限，难以适配多组分复方制剂的检测需求。

现有报道中甘油、醋酸及亚硫酸氢钠检测方法还包括气相色谱法、离子色谱法等。气相色谱法检测需样品前处理，以内标定量，分析过程需要已知浓度标准品校正^［6-9］^。离子色谱法结合脉冲安培检测（PAD）/抑制电导检测，在热不稳定化合物的分析应用中具有明显优势，但复杂样品基质会影响目标物的保留时间、峰形和检测灵敏度，而且需要配备特定设备检测^［10-12］^。

考虑到复方氨基酸注射液中成分较多，氨基酸组分容易干扰待测物的分析。国家药典委员会2024年修订了复方氨基酸注射液（14AA）药品标准并公示了该品种标准草案，新增了这3种成分的检查项，甘油采用高碘酸钠氧化后电位滴定法，亚硫酸氢钠采用与碱性品红反应后紫外-可见分光光度法，醋酸则通过液相色谱-紫外末端吸收法开展检测。但这3种方法均涉及人工操作步骤多、仪器种类多及灵敏度较低等问题，且无法实现同时测定3种成分，不适用于大批量样品的放行检验。

根据《已上市化学仿制药（注射剂）一致性评价技术要求（征求意见稿）》的要求，注射剂仿制药中的辅料（抑菌剂、缓冲剂、抗氧剂等）浓度和用量应满足相关限度要求。因此建立科学可行的含量测定方法对产品的质量控制和处方分析具有重要意义^［2，13，14］^。

离子排斥色谱通常采用总体磺化的高容量阳离子交换树脂色谱柱，在分离过程中，固定相表面形成一种半透膜，离解型离子受Donnan排斥作用无法穿过膜层，而未离解型分子可透过膜层，与固定相发生相互作用而产生保留，从而将目标物进行分离^［15-19］^。本文采用离子排斥色谱技术，建立了同时测定复方氨基酸注射液（14AA）中甘油、醋酸及亚硫酸氢钠的含量测定方法，该方法可有效排除14种高浓度氨基酸对测定的干扰，且不需要专用仪器，专属性强，通用性高，有助于全面考察注射液的稳定性和安全性，可为复方氨基酸注射液质量控制方法提供参考依据。

## 1 实验部分

### 1.1 仪器、试剂与材料

UltiMate-3000高效液相色谱仪（配备Chromeleon 7.2版网络数据系统，Thermo Fisher公司），十万分之一电子天平（XP205型，Mettler Toledo公司）；Milli-Q超纯水器（IQ7000，Merck Millipore公司）。

硫酸（北京化工厂，批号：20180604，质量分数：95.0%~98.0%）；Milli-Q超纯水（实验室自制，大于18.2 MΩ·cm）；0.22 μm水相针式滤器（材质：聚醚砜；来源：上海安谱实验科技股份有限公司）；甘油对照品（百灵威公司，批号：LF70Y162，纯度：99.7%），无水乙酸钠对照品（Thermo公司，批号：059326，纯度：99.9%），亚硫酸氢钠对照品（Sigma公司，批号：MKCP4836，纯度：64.4%）；供试品：复方氨基酸注射液（14AA）（市售品，批号：K25041301）。

### 1.2 溶液配制

空白溶剂：水。

阴性基质溶液：按处方比例配制仅含有14种氨基酸，不含甘油、醋酸及亚硫酸氢钠的溶液。

混合对照品储备液：精密称取甘油600 mg、无水醋酸钠 25.0 mg、亚硫酸氢钠对照品10.0 mg置于10 mL量瓶中，加水溶解并稀释至刻度，摇匀。

混合对照品溶液：精密移取混合对照品储备液5 mL，置于10 mL量瓶中，用水稀释至刻度，摇匀。

供试品溶液：取复方氨基酸注射液（14AA）过滤，取续滤液，即得。

### 1.3 色谱条件

色谱柱：Xtimate Sugar‐H（300 mm×7.8 mm，5 μm）；等度洗脱，流动相：7.5 mmol/L硫酸溶液，流速0.5 mL/min，柱温65 ℃，检测波长200 nm，进样量10 μL。

## 2 结果与讨论

### 2.1 色谱条件优化

本实验中以稀硫酸为流动相，考察不同柱温（30、50、55、60、65、70 ℃）条件下待测物的分离情况。结果显示，柱温在65 ℃时，甘油及醋酸与相邻峰的分离度最佳（如图1a）。同时，考察了流动相中硫酸浓度（5.0、7.5、10.0、12.5、15.0、20.0、25.0 mmol/L）对分离效果的影响，结果显示在硫酸浓度为7.5 mmol/L条件下，甘油及醋酸与相邻峰的分离度最佳（如图1b）。

**图 1:**
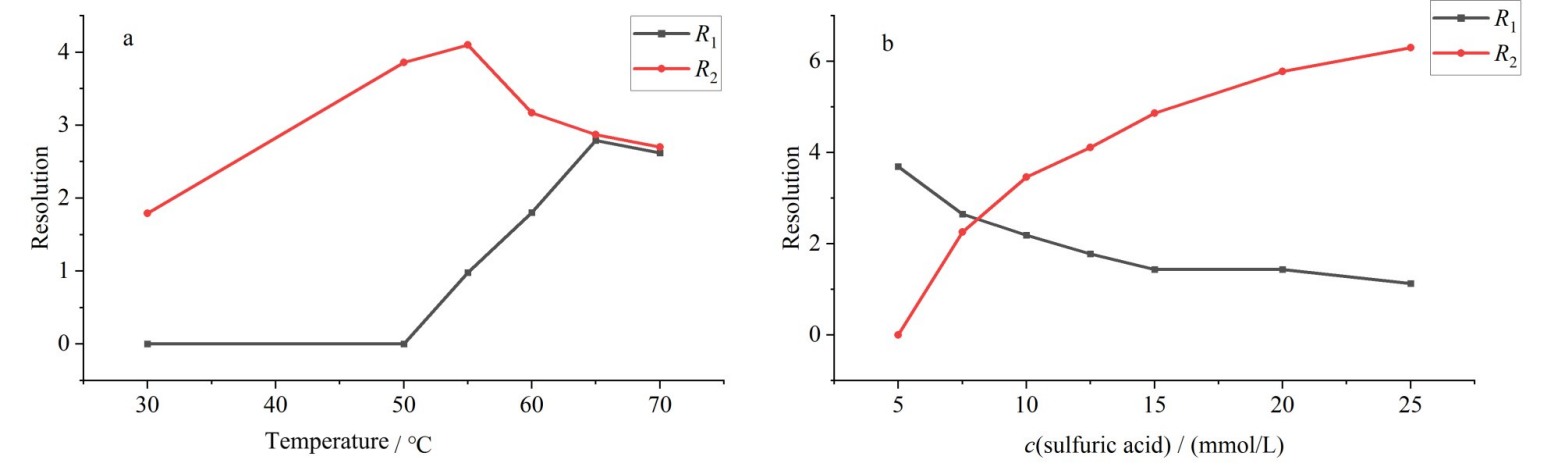
（a）柱温和（b）流动相浓度对分离度的影响

### 2.2 检测器的选择

甘油、醋酸及亚硫酸氢根缺乏强紫外发色团，仅在紫外区末端有吸收。本文建立的方法使用紫外检测器，采用末端吸收波长（195、200、205 nm）开展实验优化，经对流动相体系进行对比考察，在200 nm波长条件下噪声低，基线平稳，灵敏度高，能较好地满足实验测定要求。紫外检测器稳定性高、普适性高，可有效减轻对特殊检测器的依赖。

### 2.3 方法学验证

#### 2.3.1 专属性试验

取1.2节的空白溶剂、阴性基质溶液、混合对照品溶液和供试品溶液，按1.3节色谱条件进样测定，记录色谱图，结果如图2所示。在该色谱条件下，甘油在15.78 min出峰，醋酸在17.18 min出峰，亚硫酸根在18.23 min出峰，理论塔板数以甘油峰计为26 195，且与其他峰分离度良好，空白溶剂、阴性基质溶液和供试品溶液中其他成分对甘油、醋酸、亚硫酸根的测定无干扰，表明该方法专属性良好。

**图 2:**
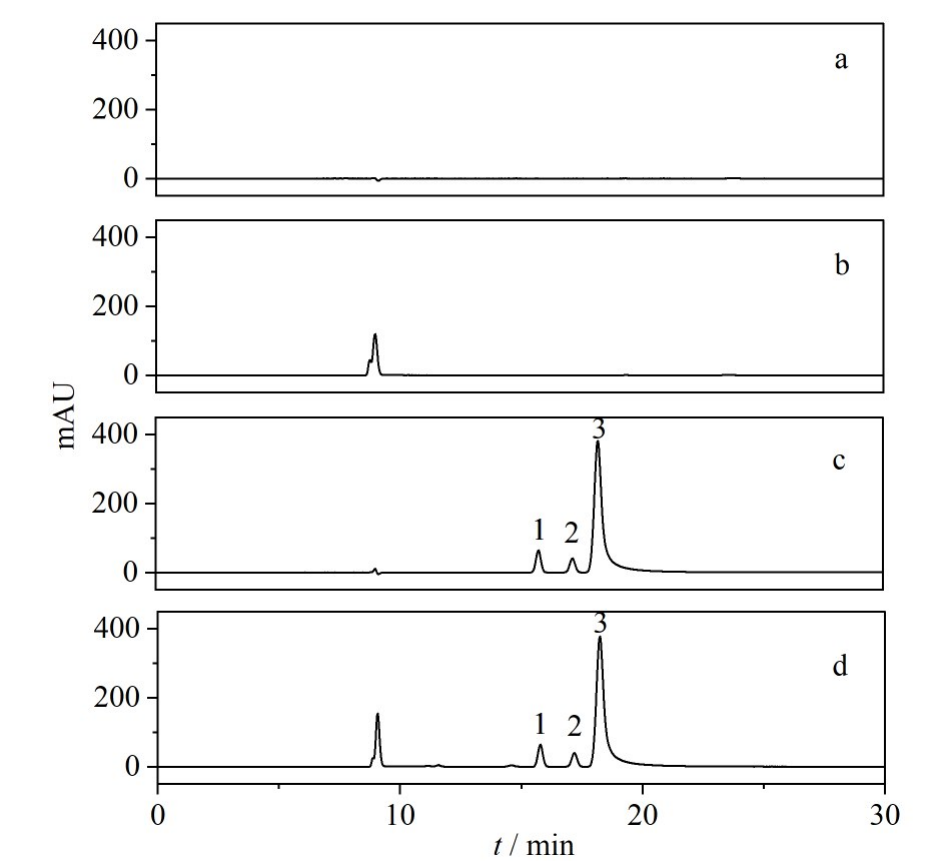
专属性试验色谱图

#### 2.3.2 强制降解试验

取复方氨基酸注射液（14AA） 2 mL置于10 mL量瓶中，平行制备6份，分别进行未破坏、强酸降解（1 mol/L盐酸溶液1 mL，破坏2 h）、强碱降解（1 mol/L氢氧化钠溶液1 mL，破坏2 h）、氧化降解（3%过氧化氢溶液1 mL，放置1 h）、高温降解（沸水浴加热2 h）和光照降解（紫外光365 nm下照射48 h），酸碱降解后进行中和，用水稀释至刻度，摇匀。取各强制降解后供试溶液按1.3节色谱条件进样测定，结果显示，在各降解条件下，供试品溶液中甘油、醋酸较为稳定，亚硫酸根在氧化降解和光降解条件下极不稳定，易进一步氧化生成硫酸根，各降解杂质不会对甘油、醋酸、亚硫酸根的测定产生干扰，结果如图3所示。

**图 3:**
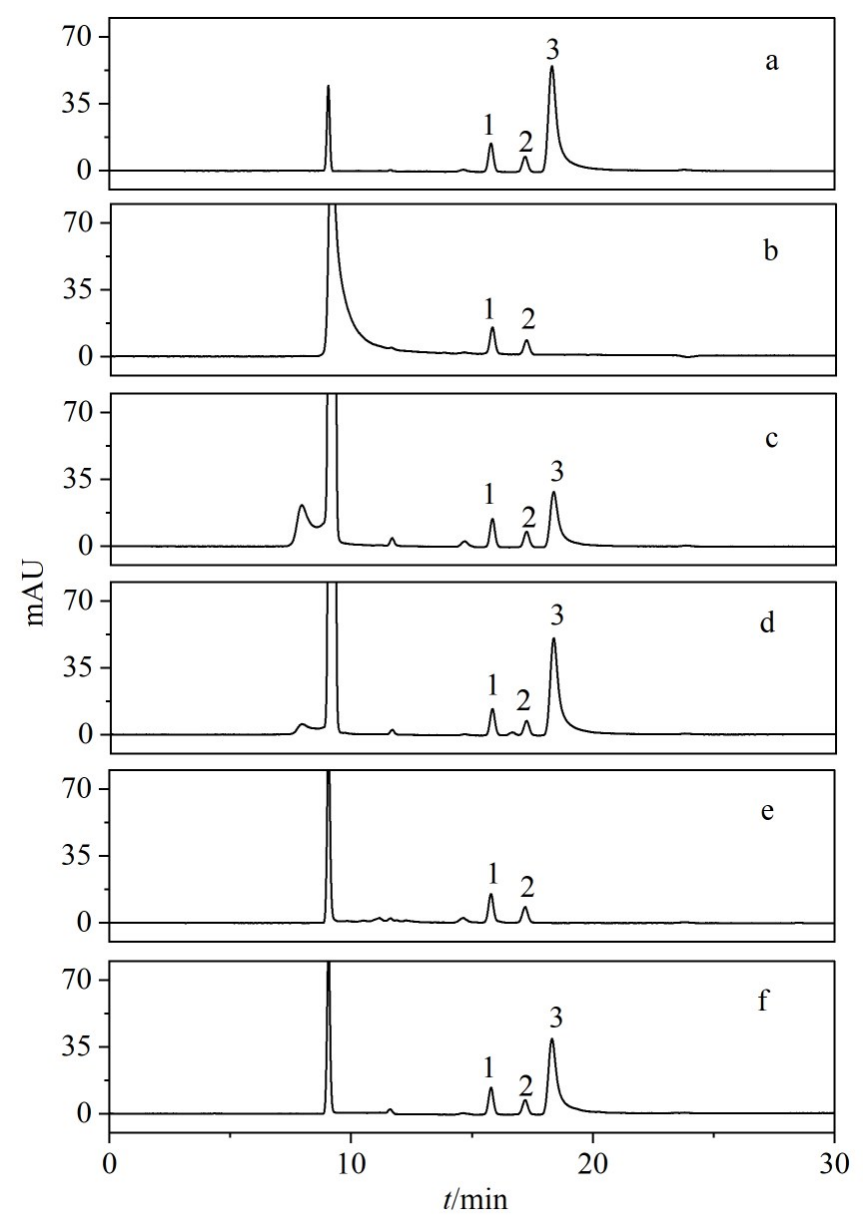
强制降解试验色谱图

#### 2.3.3 线性关系、定量限和检出限

准确称取甘油、无水醋酸钠、亚硫酸氢钠对照品适量，加水溶解并配制成质量浓度分别为甘油1.197~59.87 mg/mL、醋酸0.036 08~1.804 mg/mL、亚硫酸根0.000 791 6~0.633 3 mg/mL的系列溶液。按1.3节色谱条件进样测定，以峰面积（*Y）*对质量浓度（*X）*进行线性回归，得到回归曲线、相关系数及线性范围。将对照品溶液进行稀释，以*S/N*≥10时测得的浓度为定量限（LOQ），以*S/N*≥3时测得的浓度为检出限（LOD）。结果见表1。

**表 1:** 甘油、醋酸、亚硫酸根的线性范围、回归方程、相关系数、检出限和定量限

Component	Linear range/（mg/mL）	Regression equation	*r*	LOQ/（μg/mL）	LOD/（μg/mL）
Glycerol	1.197-59.87	*y*=0.4397*x*+0.2317	0.9998	374.5	187.3
Acetate acid	0.03608-1.804	*y*=11.594*x*+0.1234	0.9999	31.00	10.33
Sulfite	0.0007916-0.6333	*y*=580.18*x*-5.5681	0.9996	0.7543	0.3771

*y*： peak area； *x*： mass concentration， mg/mL.

#### 2.3.4 精密度和重复性试验

取1.2节对照品溶液，按照1.3节色谱条件进样分析，结果显示，连续测定6次，甘油峰面积的RSD为0.31%，醋酸峰面积的RSD为0.20%，亚硫酸根峰面积的RSD为0.42%，表明仪器的精密度良好。

精密量取复方氨基酸注射液（14AA），过滤，取续滤液，平行制备6份。按照1.3节色谱条件进样分析。6份供试品溶液分别进样，按外标法计算甘油含量的RSD为0.54%，醋酸含量的RSD为0.70%，亚硫酸根含量的RSD为1.19%，表明该方法重复性良好。

#### 2.3.5 稳定性试验

取1.2节的供试品溶液和对照品溶液，按1.3节色谱条件，室温放置分别于0、2、4、6、8、12、18、24 h进行测定。结果显示，供试品溶液放置24 h，甘油峰面积的RSD为0.64%，醋酸峰面积的RSD为0.67%，亚硫酸根峰面积的RSD为2.22%；对照品溶液室温放置24 h，甘油峰面积的RSD为0.67%，醋酸峰面积的RSD为0.49%，表明供试品溶液和对照品溶液在室温条件下24 h，甘油和醋酸稳定性良好；而亚硫酸根在室温放置8 h，峰面积的RSD为2.88%，室温放置24 h，峰面积的RSD为6.65%，表明亚硫酸根在8 h内稳定性基本满足要求，但在室温中继续放置后，亚硫酸盐会进一步氧化为硫酸，建议样品临用新制，并在8 h内完成测定。

#### 2.3.6 回收率试验

精密称取甘油1 500 mg、无水醋酸钠62.50 mg、亚硫酸氢钠对照品23.43 mg，置于25 mL量瓶中，加水溶解并稀释至刻度，摇匀，作为对照品储备液S1。精密量取对照品储备液S1 2.5 mL置于5 mL量瓶中，用水稀释至刻度，摇匀，即得对照品溶液S2。精密量取复方氨基酸注射液（14AA）5 mL置于10 mL量瓶中，分别加入对照品储备液S1 1.5、2.5、3.5 mL，用流动相稀释至刻度，摇匀，滤过，取续滤液，低、中、高浓度每个浓度配制3份。按照1.3节色谱条件进样分析，计算平均回收率和RSD。

结果（见表2）显示，在标示浓度的80%～120%范围内，甘油低、中、高浓度点的平均回收率为99.21%~100.8%，RSD为0.28%~0.53%（*n*=3）；醋酸低、中、高浓度点的平均回收率为99.25%~100.7%，RSD为0.09%~0.50%（*n*=3）；亚硫酸根低、中、高浓度点的平均回收率为100.3%~102.6%，RSD为0.20%~0.86%（*n*=3），表明该方法准确度良好。

**表 2:** 甘油、醋酸、亚硫酸根在复方氨基酸注射液（14AA）中的加标回收率（*n*=3）

Component	Background/（mg/mL）	Added/（mg/mL）	Found/（mg/mL）	Recovery/%	RSD/%
Glycerol	15.14	8.930	24.14	100.8	0.53
	15.14	14.88	30.00	99.92	0.28
	15.14	20.84	35.81	99.21	0.48
Acetateacid	0.4520	0.2732	0.7236	99.25	0.50
	0.4520	0.4554	0.9078	100.0	0.09
	0.4520	0.6375	1.094	100.7	0.11
Sulfite	0.1352	0.09243	0.2279	100.3	0.86
	0.1352	0.1540	0.2933	102.6	0.20
	0.1352	0.2157	0.3536	101.3	0.35

#### 2.3.7 耐用性

考察流速、柱温以及流动相浓度对甘油、醋酸、亚硫酸根含量测定的影响。按1.2节方法制备对照品溶液和供试品溶液，按1.3节色谱条件进样测定。当流速（0.5±0.05） mL/min、柱温（65±2） ℃以及流动相浓度（7.5±0.5） mmol/L发生变化时，甘油含量的RSD为0.94%（*n*=7）、醋酸含量的RSD为0.42%（*n*=7）、亚硫酸根含量的RSD为0.40%（*n*=7）。结果显示上述条件变化对甘油、醋酸及亚硫酸根含量测定几乎无影响。

### 2.4 实际样品测定

取复方氨基酸注射液（14AA），按照1.2节制备各溶液，按1.3节色谱条件进样测定，采用外标法进行甘油、醋酸、亚硫酸根含量测定，实验结果表明测定的甘油含量为30.31 mg/mL，为标示量的101.0%；醋酸含量为0.8828 mg/mL（无标示量）；亚硫酸根含量为0.268 0 mg/mL，为标示量的53.6%。

## 3 结论

本研究建立了离子排斥色谱同时测定复方氨基酸注射液（14AA）中甘油、醋酸、亚硫酸氢钠的方法。该方法可有效排除14种氨基酸的干扰，以稀硫酸作为流动相，不需要有机试剂，环保，专属性强，准确度高，为复方氨基酸注射液（14AA）中甘油、醋酸、亚硫酸氢钠质量控制提供了理论依据。
